# Risk Factors and Predictive Models for Peripherally Inserted Central Catheter Unplanned Extubation in Patients With Cancer: Prospective, Machine Learning Study

**DOI:** 10.2196/49016

**Published:** 2023-11-16

**Authors:** Jinghui Zhang, Guiyuan Ma, Sha Peng, Jianmei Hou, Ran Xu, Lingxia Luo, Jiaji Hu, Nian Yao, Jiaan Wang, Xin Huang

**Affiliations:** 1 Teaching and Research Section of Clinical Nursing Xiangya Hospital of Central South University Changsha, Hunan China; 2 Xiangya School of Nursing Central South University Changsha, Hunan China; 3 National Clinical Research Center for Geriatric Diseases Xiangya Hospital Central South University Changsha, Hunan China; 4 Vascular Access Department Hainan Provincial People's Hospital Hainan China; 5 Department of Nursing Affiliated Hospital of Qinghai University Qinghai China

**Keywords:** cancer, PICC, unplanned extubation, predictive model, logistic, support vector machine, random forest

## Abstract

**Background:**

Cancer indeed represents a significant public health challenge, and unplanned extubation of peripherally inserted central catheter (PICC-UE) is a critical concern in patient safety. Identifying independent risk factors and implementing high-quality assessment tools for early detection in high-risk populations can play a crucial role in reducing the incidence of PICC-UE among patients with cancer. Precise prevention and treatment strategies are essential to improve patient outcomes and safety in clinical settings.

**Objective:**

This study aims to identify the independent risk factors associated with PICC-UE in patients with cancer and to construct a predictive model tailored to this group, offering a theoretical framework for anticipating and preventing PICC-UE in these patients.

**Methods:**

Prospective data were gathered from January to December 2022, encompassing patients with cancer with PICC at Xiangya Hospital, Central South University. Each patient underwent continuous monitoring until the catheter’s removal. The patients were categorized into 2 groups: the UE group (n=3107) and the non-UE group (n=284). Independent risk factors were identified through univariate analysis, the least absolute shrinkage and selection operator (LASSO) algorithm, and multivariate analysis. Subsequently, the 3391 patients were classified into a train set and a test set in a 7:3 ratio. Utilizing the identified predictors, 3 predictive models were constructed using the logistic regression, support vector machine, and random forest algorithms. The ultimate model was selected based on the receiver operating characteristic (ROC) curve and TOPSIS (Technique for Order Preference by Similarity to Ideal Solution) synthesis analysis. To further validate the model, we gathered prospective data from 600 patients with cancer at the Affiliated Hospital of Qinghai University and Hainan Provincial People’s Hospital from June to December 2022. We assessed the model’s performance using the area under the curve of the ROC to evaluate differentiation, the calibration curve for calibration capability, and decision curve analysis (DCA) to gauge the model’s clinical applicability.

**Results:**

Independent risk factors for PICC-UE in patients with cancer were identified, including impaired physical mobility (odds ratio [OR] 2.775, 95% CI 1.951-3.946), diabetes (OR 1.754, 95% CI 1.134-2.712), surgical history (OR 1.734, 95% CI 1.313-2.290), elevated D-dimer concentration (OR 2.376, 95% CI 1.778-3.176), targeted therapy (OR 1.441, 95% CI 1.104-1.881), surgical treatment (OR 1.543, 95% CI 1.152-2.066), and more than 1 catheter puncture (OR 1.715, 95% CI 1.121-2.624). Protective factors were normal BMI (OR 0.449, 95% CI 0.342-0.590), polyurethane catheter material (OR 0.305, 95% CI 0.228-0.408), and valved catheter (OR 0.639, 95% CI 0.480-0.851). The TOPSIS synthesis analysis results showed that in the train set, the composite index (Ci) values were 0.00 for the logistic model, 0.82 for the support vector machine model, and 0.85 for the random forest model. In the test set, the Ci values were 0.00 for the logistic model, 1.00 for the support vector machine model, and 0.81 for the random forest model. The optimal model, constructed based on the support vector machine, was obtained and validated externally. The ROC curve, calibration curve, and DCA curve demonstrated that the model exhibited excellent accuracy, stability, generalizability, and clinical applicability.

**Conclusions:**

In summary, this study identified 10 independent risk factors for PICC-UE in patients with cancer. The predictive model developed using the support vector machine algorithm demonstrated excellent clinical applicability and was validated externally, providing valuable support for the early prediction of PICC-UE in patients with cancer.

## Introduction

Peripherally inserted central catheters (PICCs) are commonly used in patients with cancer who need long-term chemotherapy and supportive care therapy [1]. PICCs can effectively minimize vascular irritation caused by chemotherapy drugs, thereby preventing extravasation and the necessity for repeated punctures [2,3]. However, PICCs also have their share of disadvantages. One significant issue is the occurrence of unplanned extubation (UE) during PICC placement, which can be both frequent and severe [4]. PICC-UE occurs when the catheter needs to be withdrawn prematurely due to severe complications or accidental dislodgment resulting from patient or operator factors [4,5]. The incidence rates for PICC-UE range from 2.5% to 40.7% [6]. The occurrence of PICC-UE poses a significant risk to patients with cancer. It not only delays chemotherapy, prolongs hospitalization, and increases the financial burden on their families but also impacts the patients’ quality of life and, in some cases, even threatens their lives [7].

Previous studies primarily focused on risk factors for PICC-related complications. These complications can be associated with a variety of factors, including (1) patient-related factors, such as critically ill bedridden patients, age, and immunity [8,9]; operator-related factors, such as puncture times, professional skills, and the use of visualization technology [10-12]; catheter-related factors, such as catheter material, catheter lumen, and catheter diameter [13-15]; and treatment process–related factors, such as chemotherapy, radiotherapy, different drug types, and other aspects [16-18]. However, there is limited research on the risk factors for PICC-UE. Existing studies have primarily centered on accidental dislodgment of ventilator tubes [4,19], with insufficient attention paid to PICC-UE. Therefore, it is imperative to identify PICC-UE risk factors and develop predictive models in patients with cancer to enhance the safety of PICC usage.

To mitigate the adverse effects of PICC-UE, a promising strategy is to identify high-risk patients and offer appropriate advice for extended catheter usage. While risk prediction models for UE have been developed for intensive care unit (ICU) patients with ventilator tracheal intubation [20,21], there are no studies or models that can identify high-risk patients for PICC-UE. Lee et al [20] developed a risk assessment tool for evaluating UE of the endotracheal tube, while Hur et al [21] used 8 years of data to build a predictive model for UE using various machine learning (ML) algorithms. While both models exhibited high sensitivity and specificity, they were designed for predicting UE in ventilator tube cases.

ML algorithms are adept at extracting key features from complex data sets and are increasingly used in diagnosing and prognosticating various diseases [22]. In the context of PICC-related complications, previous studies have used ML techniques to assess risk [23,24]. Badheka et al [23] identified high-risk predictors of catheter-related thrombosis in infants under 1 year using conventional and neural network methods. Conversely, Liu et al [24] developed a predictive model for PICC-related vein thrombosis in patients with cancer using the least absolute shrinkage and selection operator (LASSO) and random forest (RF) algorithms, which exhibited impressive performance. However, as far as we know, no specific research on ML for PICC-UE in patients with cancer has been conducted yet.

This study aimed to identify PICC-UE risk factors in patients with cancer, develop and validate ML-based predictive models for PICC-UE, and promote early intervention to reduce its incidence and enhance patients’ quality of life. This study represents the first attempt to identify high-risk PICC-UE patients and serves as a valuable reference for future research and medical decision-making. We followed the *Guidelines for Developing and Reporting Machine Learning Predictive Models in Biomedical Research* [25] to report our study.

## Methods

### Study Design and Participants

This study used data from Xiangya Hospital of Central South University to build a predictive model for PICC-UE. Prospective data were collected from various hospital systems from January 1, 2022, to December 31, 2022, including the infusion system, the in-hospital Hitech electronic case system, and the PICC catheter integrated case management system. We utilized all available data to identify independent risk factors. The entire data set was divided into a train set and a test set using a 7:3 ratio through the random number table method. The train set was used for model construction, while the test set was used for internal validation. We collected data from the Affiliated Hospital of Qinghai University and Hainan Provincial People’s Hospital to perform additional validation of the model between June 1, 2022, and December 31, 2022. The external validation data were sourced from different hospitals and were independent of the data used for model construction.

Inclusion criteria were as follows: (1) pathological diagnosis of oncology; (2) availability of PICC catheterization information; and (3) voluntary participation with informed consent. Exclusion criteria were as follows: (1) patients or caregivers unable to cooperate with the investigation; (2) patients who missed visits before catheter removal; (3) incomplete data collection; and (4) abnormal values that affect judgment.

### Sample Size and Sampling

We used the sample size formula designed for cohort studies to calculate the minimum number of PICC-UE cases needed. Then, we determined the sample size required to prospectively enroll patients with cancer with PICC insertions for this study based on the PICC-UE incidence. We set α=.05 and β=.10 and obtained μα/2=1.96 and μβ=1.28.

Previous studies [4,5] have identified multiple risk factors for PICC-UE, and among these risk factors, thrombosis had the largest minimum sample size requirement for the case group. In the group without PICC-UE, the incidence of thrombosis was 8.9% (22/247; *P*_0_=.09), whereas in the group with PICC-UE, it was 27% (12/44; *P*_1_=.27). Hence, this study’s case group (UE cases) requires a minimum sample size of 164. The incidence of PICC-UE is reported as 9% (11/121) [6]. Based on this value, the initial sample size needed for a prospective study was 2448. After accounting for the possibility of missed visits and increasing the sample size by 20%, the required sample size is at least 2937.

### Instruments

The follow-up data collection schedule and clinical data collection form for this study were established through a literature review [4-19,23,24], semistructured interviews, and research group discussions.

The study investigators enrolled eligible participants who provided informed consent into a cancer whole-course management system. One-to-one follow-up through WeChat (Tencent Holdings Ltd.) was established, with follow-ups scheduled in advance. Patients were reminded to contact the investigators immediately in case of any catheter-related abnormalities. Collected data included observations of catheter patency; signs of redness, swelling, and pain in the extremity at the insertion site; blood and fluid leakage at the puncture site; catheter prolapse and its length; and any other abnormalities. Additionally, PICC-UE occurrences were monitored, and their time and reasons were recorded. Follow-up visits were conducted on the day of placement, as well as on days 1, 7, 14, 21, and every 21 days thereafter.

A total of 33 relevant factors were collected for data analysis, categorized as follows: (1) general information (gender, age, tumor type, education, BMI [calculated using height and weight], alcohol history, mental status, cooperation, and physical mobility); (2) medical history (history of deep vein thrombosis, history of central venous placement, diabetes, hypertension, cardiovascular disease, hyperlipidemia, and surgical history); (3) laboratory indicators (D-dimer concentration and fibrinogen concentration); (4) therapy schedule (radiotherapy treatment, targeted therapy, surgical treatment, anticoagulation, chemotherapy treatment, and hyperosmolar drugs); and (5) placement information (limb on the side of placement, puncture method, puncture times, catheter gauge, catheter lumen, catheter material, presence of a valve, high-pressure–resistant catheter, and catheter indwelling time). All variables were collected through observation using patient IDs and case numbers as the indexes. Data were obtained from the hospital’s Safe Infusion System (SIS) database and the Hitech electronic case system. Detailed explanations of the corresponding variables can be found in Multimedia Appendix 1.

Criteria for PICC-UE, based on previous studies [4,5], were as follows: (1) a patient who still requires a PICC catheter, but experiences early extubation due to severe complications; and (2) a patient who still requires a PICC catheter, but experiences accidental catheter dislodgment due to patient or operator factors. PICC-UE serves as the primary outcome of this study.

### Risk Factors Identification and Model Development

We reviewed the prospective data collected and categorized continuous variables, such as age, into 6 groups: “0-11,” “12-18,” “19-35,” “36-59,” “60-75,” and “≥76.” The variables *height* and *weight* were used to calculate BMI. D-dimer concentration and fibrinogen concentration values were converted into *high* or *low* categories. Missing values in the vector data were removed.

We conducted a univariate analysis of the overall data to identify variables with 2-sided statistical significance (*P*<.05). Following a literature review and expert consultations, we used the LASSO regression algorithm to include clinically significant variables. The selected variables underwent multifactorial analysis to identify independent risk factors for PICC-UE in patients with cancer.

The model was constructed using prospective data from Xiangya Hospital of Central South University. Data order was randomized using a shuffling algorithm for even distribution. The data were then split into a train set and a test set at a ratio of 7:3 using the random number table method. The overall data were used for independent risk factor screening, the train set for model construction, and the test set for internal model validation. The risk prediction models were constructed using the train set, incorporating prescreened independent risk factors. In this study, 3 ML algorithms, namely, logistic regression (LR), support vector machine (SVM), and RF, were selected to build risk prediction models for PICC-UE in patients with cancer.

We compared these models using the area under the receiver operating characteristic (ROC) curve (AUC) and the TOPSIS (Technique for Order Preference by Similarity to Ideal Solution) method [26]. AUC assesses the predictive power of the PICC-UE model, while the model’s superiority was evaluated based on the Composite Index (Ci) value in the TOPSIS method. The model with the highest AUC and Ci values was considered optimal for predicting PICC-UE and selected as the best model.

### Validation and Model Performance Evaluation

Data from June 2022 to December 2022 from Qinghai University Hospital and Hainan Provincial People’s Hospital were used for external validation. The collected data were randomized using a shuffling algorithm for even distribution. The optimal model was assessed for discrimination, calibration, and clinical applicability.

Discrimination assesses the model’s ability to distinguish between high and low PICC-UE risk in the cancer population, which we evaluated using the AUC. Calibration indicates the degree of agreement between the predicted and actual results. The calibration of the model was assessed using the Hosmer-Lemeshow test with a calibration curve [27]. Clinical applicability, which gauges the diagnostic accuracy of the model in clinical use, was evaluated using decision curve analysis (DCA) [21]. Additionally, model performance was measured using sensitivity, specificity, positive predictive value, negative predictive value [24], and AUC.

### Ethical Considerations

The study was approved by the Hospital Ethics Review Committee (approval number 202204210). We adhered to the principles of informed consent, data confidentiality, anonymity, and nonharmfulness. Written informed consent was collected, and any papers or publications based on the study data will not reveal personal information about the patients. For younger or unconscious patients who were unable to participate, data collection was facilitated by their caregivers.

### Statistical Analysis

We excluded data with missing or unusual variables from the prospective data set. Continuous variables were compared using independent-sample unpaired (2-sided) *t* tests or one-way analysis of variance (ANOVA). Categorical variables were presented as numbers and proportions and compared using the chi-square test or Fisher exact test. We collected variables with bilateral *P*<.05 statistical significance and then included variables with potential clinical significance for the LASSO algorithm based on literature analysis and expert consultation. We identified independent risk factors for PICC-UE in patients with cancer through multifactorial analysis. After consulting with experts in ML algorithms and discussions within the research group, we chose 3 ML methods to construct the study’s model: RF, SVM, and LR.

All hypothesis tests with 2-sided *P*<.05 indicated statistical significance. The “na.omit” function was used to remove missing values from the vector data. LASSO primarily used the “glmnet” package with a 10-fold orthogonal method to define the penalty function. LR, RF, and SVM were mainly implemented using “caret,” “randomForest,” “pROC,” “varImpPlot,” and “e1071,” respectively. The ROC curves were plotted using the “pROC” packet, and the Hosmer-Lemeshow test using the “hoslem.test,” “data.table,” and “plyr” data packages was used for the TOPSIS integrated analysis. The DCA decision curves were constructed using the “rms” and “rmda” packets. All the analyses were performed using R Statistical Software, version 4.1.3 (R Foundation).

## Results

### Participants Characteristics

**Figure 1:**
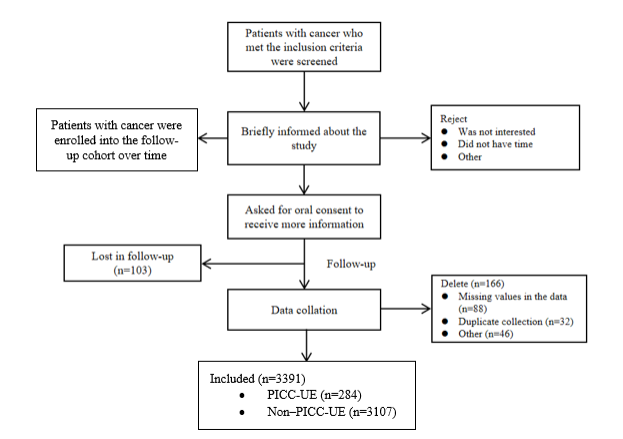
Patient recruitment flowchart.

A total of 3391 patients were included, with a sample loss rate of 7.34% (269/3660). This included 2374 in the train set and 1017 in the test set, with 284 PICC-UE cases. The study flow diagram is presented in Figure 1. Baseline participant characteristics are presented in Table 1. Importantly, there was no multicollinearity among the variables, as all variance inflation factor values were less than 5.0.

**Table 1:** Comparison of general information between the control and case groups^a^.

Variables		Non–PICC-UE^b^ (n=3107)	PICC-UE (n=284)	Chi-square/*F* test (*df*)	*P* value
**Gender, n (%)**				6.349^c^ (1)	.01^d^
	Male	1486 (47.83)	158 (55.63)		
	Female	1621 (52.17)	126 (44.37)		
**Age (years), n (%)**				14.431^c^ (5)	.01^d^
	0-11	57 (1.83)	3 (1.06)		
	12-18	73 (2.35)	5 (1.76)		
	19-35	208 (6.69)	15 (5.28)		
	36-59	1695 (54.55)	140 (49.30)		
	60-75	962 (30.96)	100 (35.21)		
	≥76	112 (3.60)	21 (7.39)		
**Tumor type** **, n (%)**				15.289^c^ (8)	.05
	Lung cancer	794 (25.56)	89 (31.34)		
	Thymic cancer breast cancer	745 (23.98)	83 (29.23)		
	Gastro-colorectal cancer	464 (14.93)	27 (9.51)		
	Hematologic tumors	407 (13.10)	30 (10.56)		
	Cervical cancer	187 (6.02)	16 (5.63)		
	Head-neck tumors	146 (4.70)	8 (2.82)		
	Hepatobiliary-pancreatic tumors	93 (2.99)	9 (3.17)		
	Intracranial tumors	95 (3.06)	8 (2.82)		
	Others	176 (5.66)	14 (4.93)		
**Educational level, n (%)**				8.088^c^ (3)	.04^d^
	Illiterate primary and junior high schools	1399 (45.03)	152 (53.52)		
	Secondary and high school	861 (27.71)	69 (24.30)		
	College bachelor’s degree	788 (25.36)	60 (21.13)		
	Master’s degree doctorate	59 (1.90)	3 (1.06)		
**BMI (kg/m^2^), n (%)**				33.741^c^ (2)	<.001^d^
	<18.5	201 (6.47)	18 (6.34)		
	18.5-24.0	1945 (62.60)	131 (46.13)		
	>24.0	961 (30.93)	135 (47.54)		
**Alcohol history, n (%)**				2.123^c^ (1)	.15
	None	2739 (88.16)	242 (85.21)		
	Yes	368 (11.84)	42 (14.79)		
**Mental status, n (%)**				5.529^c^ (1)	.02^d^
	Sobriety	2930 (94.30)	258 (90.85)		
	Blurred consciousness	177 (5.70)	26 (9.15)		
**Cooperation, n (%)**				8.528^c^ (1)	.004^d^
	Cooperative	2909 (93.63)	253 (89.08)		
	Noncooperative	198 (6.37)	31 (10.92)		
**Physical mobility, n (%)**				43.276^c^ (1)	<.001^d^
	Normal	2884 (92.82)	232 (81.69)		
	Abnormal	223 (7.18)	52 (18.31)		
**History of deep vein thrombosis, n (%)**				12.799^c^ (1)	<.001^d^
	None	2909 (93.63)	250 (88.03)		
	Yes	198 (6.37)	34 (11.97)		
**History of central venous placement, n (%)**				7.724^e^ (3)	.05
	None	2720 (87.54)	233 (82.04)		
	1	235 (7.56)	29 (10.21)		
	2	107 (3.44)	15 (5.28)		
	≥3	45 (1.45)	7 (2.46)		
**Diabetes, n (%)**				13.381^c^ (1)	<.001^d^
	None	2935 (94.46)	253 (89.08)		
	Yes	172 (5.54)	31 (10.92)		
**Hypertension, n (%)**				0.149^c^ (1)	.70
	None	2875 (92.53)	261 (91.90)		
	Yes	232 (7.47)	23 (8.10)		
**Cardiovascular disease, n (%)**				3.023^c^ (1)	.08
	None	2897 (93.24)	257 (90.49)		
	Yes	210 (6.76)	27 (9.51)		
**Hyperlipidemia, n (%)**				14.841^c^ (1)	<.001^d^
	None	2864 (92.18)	243 (85.56)		
	Yes	243 (7.82)	41 (14.44)		
**Surgical history, n (%)**				21.580^c^ (1)	<.001^d^
	None	1935 (62.28)	137 (48.24)		
	Yes	1172 (37.72)	147 (51.76)		
**D-dimer concentration, n (%) (mg/dl)**				66.054^c^ (1)	<.001^d^
	≤0.5	2632 (84.71)	187 (65.85)		
	>0.5	475 (15.29)	97 (34.15)		
**Fibrinogen concentration, n (%) (mg/dl)**				10.658^c^ (2)	.005^d^
	Lower	94 (3.03)	7 (2.46)		
	Normal	2315 (74.51)	189 (66.55)		
	Higher	698 (22.47)	88 (30.99)		
**Radiotherapy treatment, n (%)**				3.464^c^ (1)	.06
	None	2828 (91.02)	249 (87.68)		
	Yes	279 (8.98)	35 (12.32)		
**Targeted therapy, n (%)**				12.042^c^ (1)	.001^d^
	None	1460 (46.99)	103 (36.27)		
	Yes	1647 (53.01)	181 (63.73)		
**Surgical treatment, n (%)**				26.409^c^ (1)	<.001^d^
	None	2483 (79.92)	190 (66.90)		
	Yes	624 (20.08)	94 (33.10)		
**Anticoagulation, n (%)**				2.759^c^ (1)	.09
	None	2903 (93.43)	258 (90.85)		
	Yes	204 (6.57)	26 (9.15)		
**Chemotherapy treatment, n (%)**				2.016^c^ (1)	.16
	None	433 (13.94)	31 (10.92)		
	Yes	2674 (86.06)	253 (89.08)		
**Hyperosmolar drugs, n (%)**				9.783^c^ (1)	.002^d^
	None	1460 (46.99)	106 (37.32)		
	Yes	1647 (53.01)	178 (62.68)		
**Limb on side of placement, n (%)**				5.718^e^ (3)	.13
	Left upper extremity	1570 (50.53)	131 (46.13)		
	Right upper extremity	1467 (47.22)	141 (49.65)		
	Left lower extremity	37 (1.19)	7 (2.46)		
	Right lower extremity	33 (1.06)	5 (1.76)		
**Puncture method, n (%)**				0.170^c^ (2)	.92
	Blind	98 (3.15)	10 (3.52)		
	Blind-MST^f^	164 (5.28)	14 (4.93)		
	Bright scan ultrasound-MST	2845 (91.57)	260 (91.55)		
**Puncture times, n (%)**				11.900^c^ (1)	.001^d^
	1	2928 (94.24)	253 (89.08)		
	Many times	179 (5.76)	31 (10.92)		
**Catheter gauge (Fr), n (%)**				3.678^e^ (3)	.26
	1.9	6 (0.19)	2 (0.70)		
	3	82 (2.64)	5 (1.76)		
	4	2847 (91.63)	260 (91.55)		
	5	172 (5.54)	17 (5.99)		
**Catheter lumen, n (%)**				0.984^c^ (1)	.32
	Single chamber	2763 (88.93)	258 (90.85)		
	Double chamber	344 (11.07)	26 (9.15)		
**Catheter material, n (%)**				13.010^c^ (1)	<.001^d^
	Silicone	1308 (42.10)	151 (53.17)		
	Polyurethane	1799 (57.90)	133 (46.83)		
**Presence of valve, n (%)**				8.821^c^ (1)	.003^d^
	None	1314 (42.29)	146 (51.41)		
	Yes	1793 (57.71)	138 (48.59)		
**Whether high-pressure–resistant catheter, n (%)**				0.791^c^ (1)	.37
	None	2435 (78.37)	229 (80.63)		
	Yes	672 (21.63)	55 (19.37)		

^a^The mean catheter indwelling time for all participants is 91.22 (SD 78.88) days, for the non–PICC-UE group is 91.26 (SD 80.15) days, and for the PICC-UE group is 90.79 (SD 78.95) days (unpaired 2-tailed *t* test =.009; *P*=.92).

^b^PICC-UE: unplanned extubation of the peripherally inserted central catheter.

^c^Chi-square test

^d^2-tailed *P*<.05.

^e^Fisher exact test.

^f^MST: modified Seldinger technique.

### Independent Risk Factor Determination

A total of 19 potential risk factors, including gender, age, and education level, were initially screened using univariate analysis. Following consultations with specialists in vascular surgery, pathology, and venous therapy, catheter lumen and central venous placement history were added. Thus, there were a total of 21 independent variables for the LASSO analysis.

**Figure 2:**
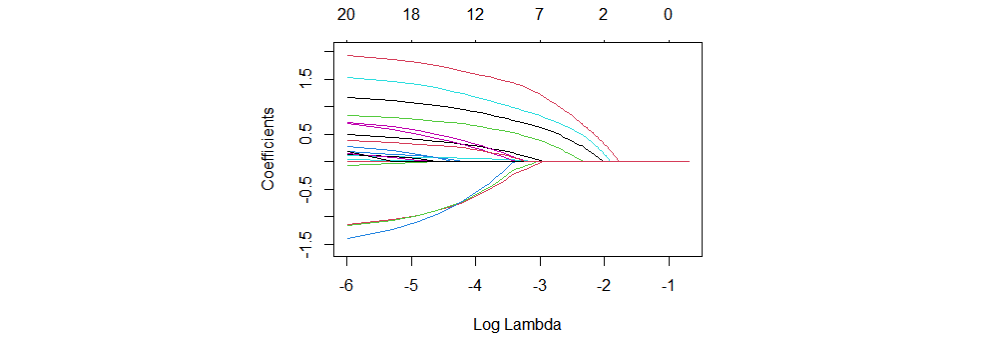
Cross-validation plot of the LASSO penalty term. LASSO: least absolute shrinkage and selection operator.

**Figure 3:**
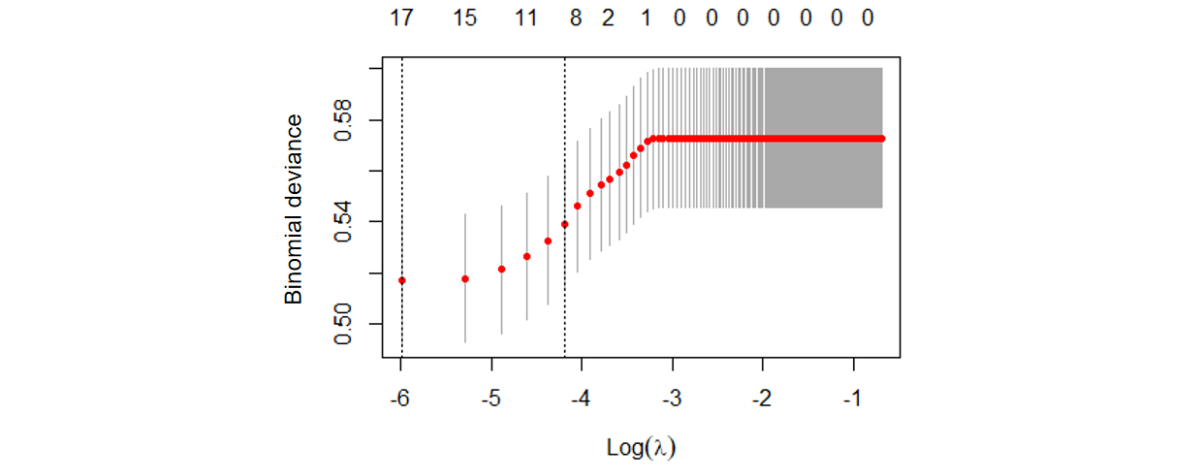
LASSO regression coefficients on the different penalty parameters. LASSO: least absolute shrinkage and selection operator.

In Figure 2, each colored line represents a variable trend that decreases as the penalty factor λ changes, resulting in the model incorporating fewer variables. In Figure 3, the dashed line on the left indicates the λ value associated with the maximum AUC and the number of features included in the model. On the right, the dashed line represents a reduction in the number of features in the model as the standard error increases by 1 to achieve the maximum AUC. The minimum error is reached at 1SE=0.013, resulting in the screening of 11 predictor variables.

The 11 predictors identified by LASSO were analyzed using conditional LR with a fixed α_in_ of 0.05 and α_out_ of 0.10, using the backward LR method. The results revealed the following independent risk factors for PICC-UE in patients with cancer (ranked by importance from high to low): impaired physical mobility (odds ratio [OR] 2.775, 95% CI 1.951-3.946), elevated D-dimer concentration (OR 2.376, 95% CI 1.778-3.176), diabetes (OR 1.754, 95% CI 1.134-2.712), surgical history (OR 1.734, 95% CI 1.313-2.290), more than 1 catheter puncture (OR 1.715, 95% CI 1.121-2.624), surgical treatment (OR 1.543, 95% CI 1.152-2.066), and targeted therapy (OR 1.441, 95% CI 1.104-1.881). Protective factors, ranked by importance from high to low, were valved catheter (OR 0.639, 95% CI 0.480-0.851), normal BMI (OR 0.449, 95% CI 0.342-0.590), and polyurethane catheter material (OR 0.305, 95% CI 0.228-0.408). Details are presented in Table 2.

**Table 2:** Multivariate analysis to identify independent risk factors.

Variables	β	Odds ratio (95% CI)	*P* value
BMI<18.5 kg/m^2^	–.340	0.712 (0.414-1.224)	.22
BMI=18.5-24.0 kg/m^2^	–.801	0.449 (0.342-0.590)	<.001
Physical mobility	1.021	2.775 (1.951-3.946)	<.001
Diabetes	.562	1.754 (1.134-2.712)	.01
Surgical history	.551	1.734 (1.313-2.290)	<.001
D-dimer concentration	.866	2.376 (1.778-3.176)	<.001
Targeted therapy	.365	1.441 (1.104-1.881)	.007
Surgical treatment	.434	1.543 (1.152-2.066)	.004
Puncture times	.540	1.715 (1.121-2.624)	.01
Catheter material	–1.188	0.305 (0.228-0.408)	<.001
Presence of valve	–.447	0.639 (0.480-0.851)	.002
Constant	–2.043	0.130 (0.087-0.193)	<.001

### Prediction Model Construction

The train set and the test set were well balanced, with no statistically significant differences in composition (*P*>.05 in all cases). Further details can be found in Multimedia Appendix 2.

The logistic predictive model was constructed using the 10 independent risk factors identified in the previous phase. The final model included 9 variables with a *χ*_8_^2^ value of 320.374 and *P*<.001. SVM modeling was performed with 10-fold cross-validation and grid search methods, autonomously determining the optimal number of vector machines and related parameters using the tune.svm function. The polynomial kernel function demonstrated the highest prediction accuracy among the 4 kernel functions. The RF predictive model for patients with cancer was constructed with a final minimum of 196 trees.

### Model Comparison and Validation

**Figure 4:**
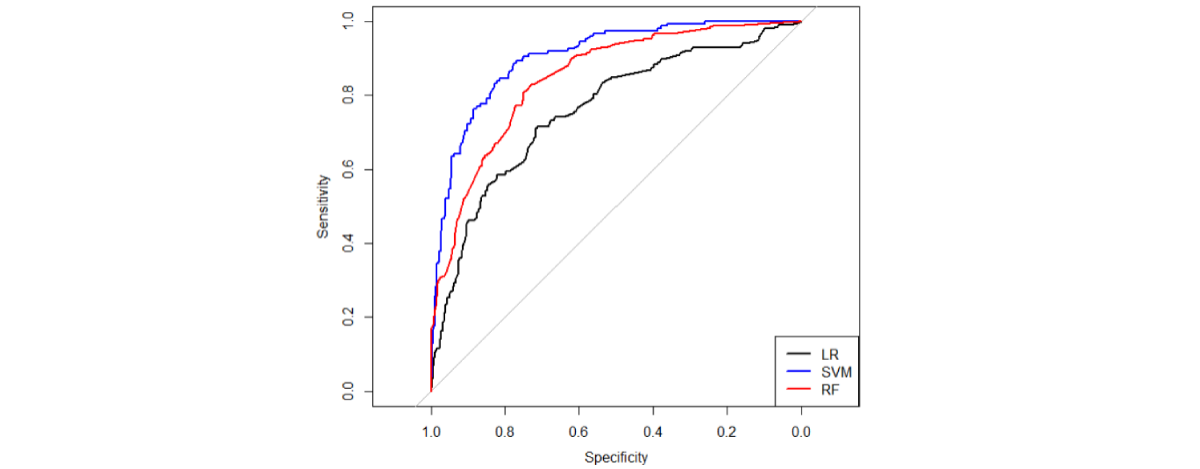
AUC (95% CI) plots of train set.

The SVM predictive model exhibited the best predictive efficacy for PICC-UE when considering AUC and Ci values together. A comparison of the ROC curves of the 3 models is presented in Figures 4 (train set) and 5 (test set). The 3 models were assessed using the TOPSIS integrated analysis in the train set and test set, as depicted in Tables 3 and 4. The RF predictive model performed the best in the train set, and the overall performance of the models is as follows: RF model>SVM model>logistic model. However, it is worth noting that the Ci value for the SVM model was 0.82, while that for the RF model was 0.85, with only a 0.03 difference. In the test set, the TOPSIS integrated analysis revealed that the SVM predictive model had the best fit, and the models ranked in terms of overall performance as SVM model>RF model>logistic model. For a visual comparison (AUC, sensitivity, specificity, accuracy, positive predictive value, and negative predictive value) of the 3 models, please refer to Figures 6 (train set) and 7 (test set). These figures demonstrate that both the SVM model and the RF model outperform the logistic model in terms of predictive effects.

**Figure 5:**
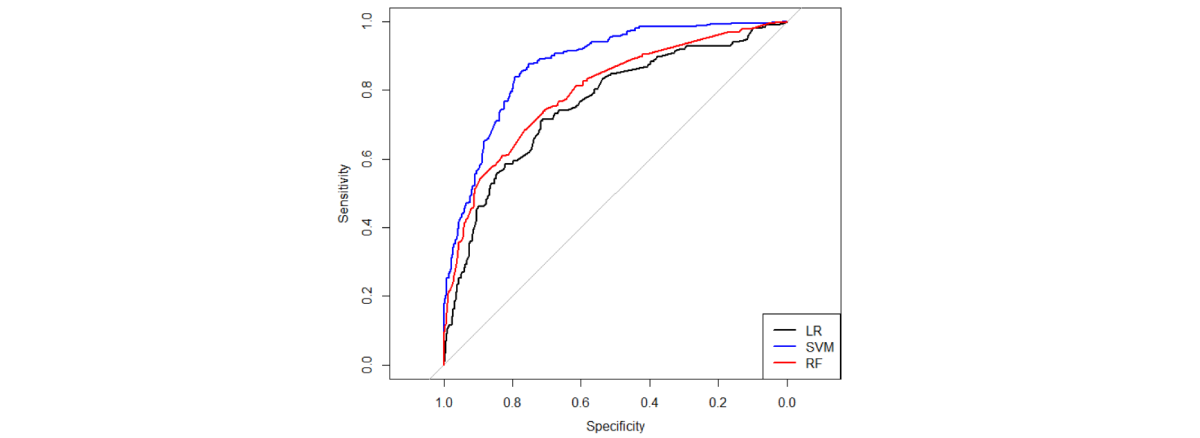
AUC (95% CI) plots of test set.

**Table 3:** Comparison of the 3 predictive models in the train set.

Model	Area under the curve (%)	Sensitivity (%)	Specificity (%)	Accuracy (%)	Positive predictive value (%)	Negative predictive value (%)	Composite Index
Logistic regression	75.8	58.38	91.91	89.13	39.52	96.06	0.00
Support vector machine	90.4	79.19	99.36	97.68	91.76	98.14	0.82
Random forest	84.7	89.34	99.44	98.61	93.62	99.04	0.85

**Table 4:** Comparison of the three predictive models in the test set.

Model	Area under the curve (%)	Sensitivity (%)	Specificity (%)	Accuracy (%)	Positive predictive value (%)	Negative predictive value (%)	Composite Index
Logistic regression	68.1	58.62	77.53	75.91	19.62	95.24	0.00
Support vector machine	87.5	88.51	99.78	98.82	97.47	98.93	1.00
Random forest	79.6	83.91	98.71	97.44	85.88	98.5	0.81

**Figure 6:**
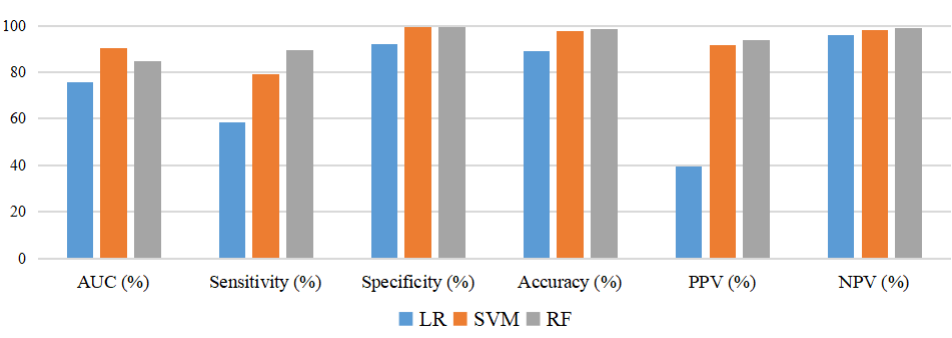
Comparison of the 3 predictive models in the train set.

**Figure 7:**
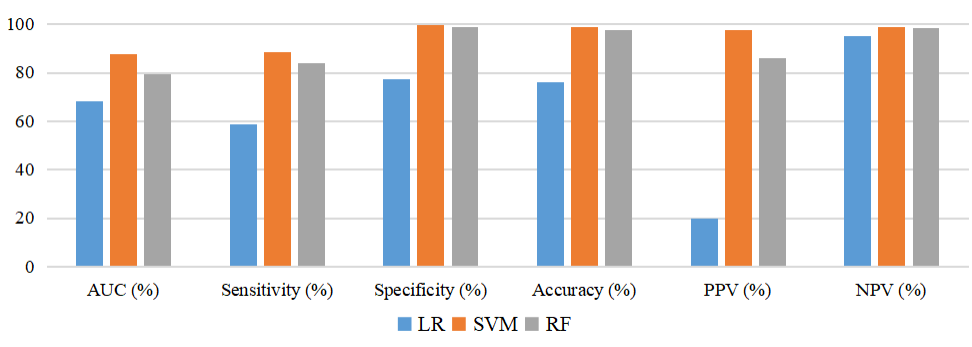
Comparison of the 3 predictive models in the test set.

**Figure 8:**
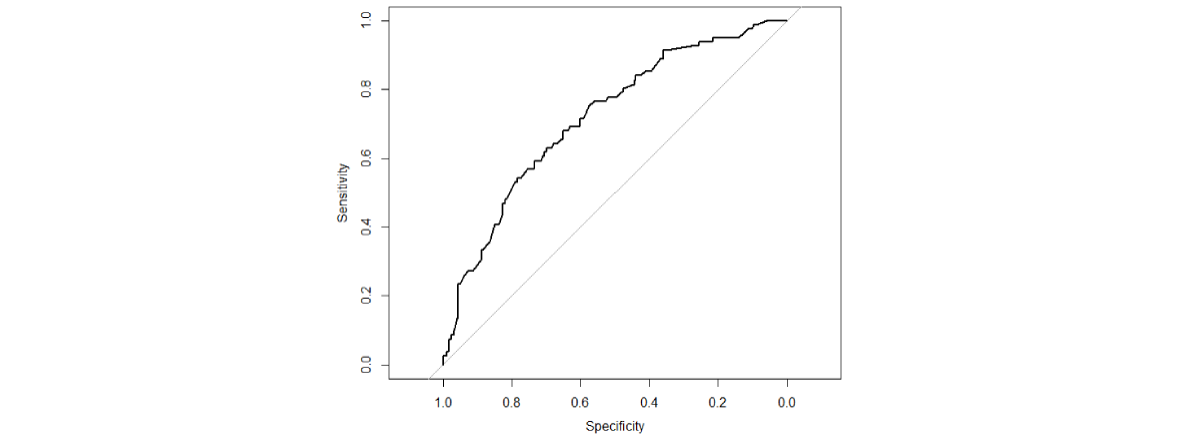
External validation of the ROC curve.

**Figure 9:**
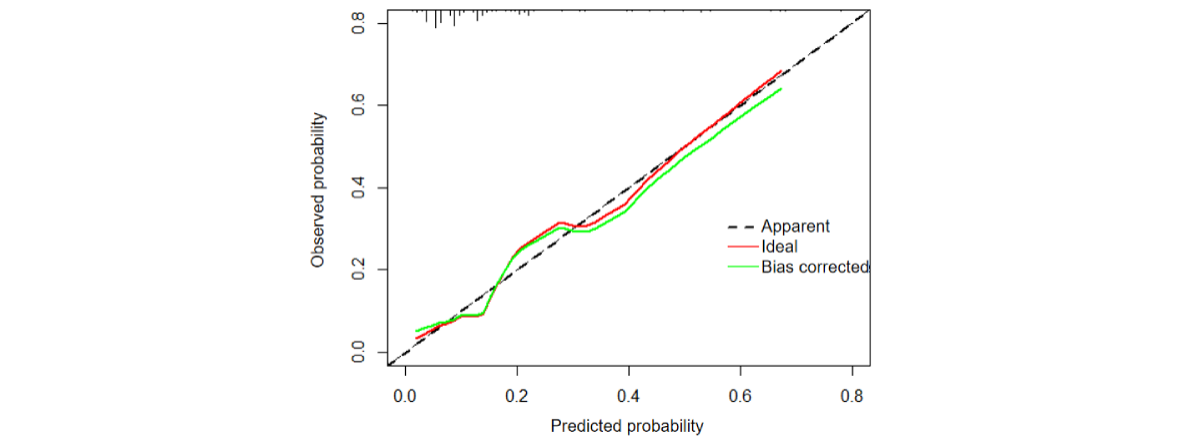
External validation of the Calibration curve.

**Figure 10:**
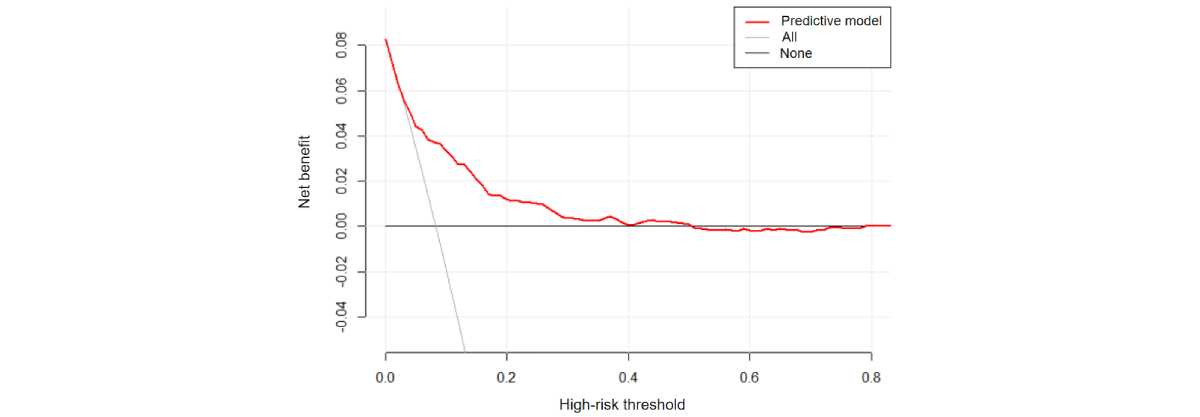
External validation of the DCA.

We assessed the performance of the best model through discrimination, calibration capability, and clinical applicability analysis. The AUC values evaluated the discrimination, and the SVM model demonstrated strong differentiation with an AUC of 0.718 for external validation (Figure 8). The Hosmer-Lemeshow test for goodness of fit resulted in *χ*_8_^2^=8.205, *P*=.06, which is greater than 0.05, indicating a well-fitting model for external validation. The calibration curve for the optimal model is presented in Figure 9. The clinical applicability of this predictive model is demonstrated by the DCA curve in Figure 10.

## Discussion

### Principal Findings

Our prospective study is a pioneering contribution to the field, being the first to develop and validate a predictive model for PICC-UE in patients with cancer that can guide decision-making without requiring extensive laboratory testing. We adhered to the *Guidelines for Developing and Reporting Machine Learning Predictive Models in Biomedical Research* for model development. Our model demonstrates outstanding performance in predicting PICC-UE in patients with cancer, achieving an AUC of 0.904 in the train set and 0.875 in the test set. Importantly, we identified 10 highly correlated independent risk factors using univariate, LASSO, and multivariate analyses to build the model, with the 3 most significant risk factors being physical mobility (*P*<.001), D-dimer concentration (*P*<.001), and diabetes (*P*=.01).

PICC-UE incidence varies, ranging from 7.5% to 22.0% in China [28,29] and from 2.5% to 40.7% in other countries [4]. Duwadi et al [30] noted a higher PICC-UE incidence in the ICU compared with other units, attributing it to the ICU environment and patient severity. Additionally, PICC-UE rates differed in studies from different regions [28,29]. In our study, the incidence of PICC-UE was 8.38% (284/3391), which is lower than in most previous studies [4,29]. This could be attributed to our hospital’s intravenous infusion therapy committee, improved standardized nurse train, rigorous quality control management, and numerous educational sessions on patient health management. These differences in incidence may also be related to variations in inclusion criteria, follow-up methods, duration, and the sample size in our study. Future prospective studies with larger, multicenter samples and extended follow-up may be necessary for further validation.

In terms of general information, medical history, and laboratory indicators, we discovered that BMI, physical mobility, diabetes, surgical history, and D-dimer concentration were linked to the occurrence of PICC-UE. In particular, patients who are overweight (BMI>24.0 kg/m^2^) [31], those with reduced physical activity [32], and individuals with diabetes prone to hypercoagulation [32] were at a higher risk of catheter thrombosis. A recent surgical trauma can also stimulate the release of a significant amount of coagulation factors to aid wound healing [33], while a prolonged period of postoperative bed rest can slow blood flow, both of which increase the risk of coagulation [34]. An elevated D-dimer level is indicative of a hypercoagulation state, with a concentration exceeding 500 μg/L signifying a high risk of thrombosis [35]. Bertoglio et al [36] demonstrated that PICC catheter thrombosis is a significant risk factor for UE. Patients with a low BMI (BMI<18.5 kg/m^2^) have compromised immunity and are prone to malnutrition, increasing their risk of catheter-related complications and the need for catheter removal [37]. Excessive physical activity increases catheter-vessel wall friction, raising the risk of bloodstream infection and early catheter dislodgment [38].

In terms of therapy schedule and placement information, we observed that targeted therapy, surgical treatment, puncture times, catheter material, and the presence of a valve were linked to the occurrence of PICC-UE. The use of targeted drugs [39] and multiple punctures [40] can lead to vascular endothelial damage, exposing subendothelial prothrombotic components, inducing platelet aggregation, contributing to catheter thrombosis, and elevating the risk of extubation [41]. Surgical treatment leading to PICC-UE aligns with the explanation of the recent surgical history mentioned earlier. Additionally, patients recovering from postoperative anesthesia are often unconscious and may inadvertently remove the catheter due to the foreign body sensation at the catheter placement site [42]. We identified a higher risk of PICC-UE associated with silicone catheters. This is attributed to the use of new high-pressure–resistant polyurethane catheters in our hospital, which incorporate a surface-active macromolecule with fluorine atom doping. This component inhibits platelet adhesion and protein procoagulation, ultimately lowering the incidence of PICC-related thrombosis [13]. Catheter valves effectively prevent blood regurgitation, reducing both catheter-related blockages and thrombosis [13].

In this study, we developed a comprehensive predictive model to assess the risk of high-risk PICC-UE in patients with cancer. The model’s performance was evaluated using the AUC as a measure of classification efficacy, and all models in our study achieved AUC values exceeding 0.7, demonstrating their strong ability to distinguish high-risk patients. After comparing the AUC and Ci values, the SVM model emerged as the optimal choice. Calibration and DCA curves confirmed the SVM model’s accuracy, stability, generalizability, and clinical applicability.

The PICC-UE predictive model for patients with cancer developed in this study using the ML algorithm offers insights for related research. In the predictor screening process, previous studies often relied on a single statistical method [43], while our approach combined univariate analysis, 10-fold cross-validation LASSO, and multivariate screening, enhancing precision and rigor. This approach resulted in the creation of a more concise and accurate predictive model through multiple rounds of variable filtering. The LASSO method effectively aggregates features, achieves dimensionality reduction, and serves as a feature screening tool, preventing issues related to covariance and overfitting [44].

This study used multiple ML algorithms to construct the predictive model, a more scientifically rigorous approach compared with using a single method alone [26]. ML algorithms are well-suited for managing high-dimensional variables and their intricate interactions, making full use of the available data [22]. The test set demonstrated superior predictive performance in forecasting PICC-UE based on the results from the train set, significantly enhancing prediction accuracy. This study compared 3 ML models and selected the best-performing one, significantly improving the model’s accuracy. We used AUC and TOPSIS methods for a comprehensive and rigorous screening of the optimal predictive model. The SVM algorithm in the optimal model robustly encompasses the data and reduces the model’s complexity through linear regression with insensitive loss functions in a high-dimensional feature space [45]. Importantly, external validation of the model using independent data demonstrated significant predictive superiority.

Our study has successfully developed a highly predictive model for the risk of PICC-UE in patients with cancer using the SVM algorithm. This model enables the development of personalized precautions for patients with cancer at a high risk of PICC-UE, such as the regular assessment of physical mobility and the provision of targeted physical activity guidance for patients with impaired physical mobility [32]. For patients with abnormal BMI, dynamic monitoring of BMI and weight adjustment through exercise and diet should be implemented [32]. Patients with diabetes require special attention [32], with routine blood tests on admission and regular monitoring of D-dimer concentrations [35] to take preventive measures against early catheter removal. For patients with a history of surgery and those undergoing surgical treatment or targeted therapy [33,34,39], close monitoring of the catheter exit site is essential. Patients should receive instructions for regular catheter maintenance and be advised to seek medical attention if they experience any discomfort. Our study concluded that patients with multiple punctures are at a higher risk of PICC-UE. It is recommended that the medical department standardizes the qualifications of PICC placement nurses and conducts regular training and assessments [40]. Furthermore, medical departments should exercise strict control over the choice of catheter materials and the presence of valves in catheters to minimize catheter-related complications and lower the incidence of PICC-UE [13].

### Limitations and Challenges

This study has some limitations. First, it did not include individual genetic data, which can be a significant factor in PICC-UE. Future studies may benefit from incorporating genetic data to improve predictive accuracy. Second, external validation was limited by a small data set, which included data from only 2 hospitals. More extensive external validation is required to thoroughly validate the predictive model. Lastly, we did not consider how the risk factors and predictive model for PICC-UE may differ among various subpopulations of patients with cancer, including different age groups, genders, and cancer stages.

Despite the limitations, our study has identified 10 independent predictors, including BMI, mobility, diabetes, surgical history, and other factors, that are significantly associated with an increased risk of PICC-UE in patients with cancer. Furthermore, our SVM predictive model has been externally validated and demonstrates excellent generalization. The optimal SVM model achieved a high accuracy of 97.68% in the train set and 98.82% in the test set, indicating excellent model fitting. The LASSO algorithm used for risk factor screening effectively prevented overfitting. Our findings can raise awareness among clinicians and patients for the early prevention and reduction of PICC-UE in high-risk cancer populations. Further prospective multicenter studies are needed to validate risk factors and establish effective UE prophylaxis interventions. Our group is in discussions with a computer company to develop a plug-in for our hospital’s electronic system. This plug-in aims to automatically capture independent risk factors for PICC-UE from patient hospitalization information. Using the optimal prediction model from this study, patients’ risk of PICC-UE is categorized into 3 levels: red (high risk), yellow (medium risk), and green (low risk). Using the color-coded cues, health care providers can implement tailored interventions for high-risk patients while offering self-monitoring guidance and health education to medium- and low-risk patients.

### Conclusions

In summary, the developed predictive model for assessing the risk of PICC-UE in patients with cancer has shown excellent discrimination, high predictive accuracy, and broad applicability across a range of risk factors. This model serves as a valuable tool for the early identification of high-risk patients and holds promise for clinical implementation.

## Appendix

Multimedia Appendix 1 Definitions for the factors examined in the models.

Multimedia Appendix 2 Detailed comparison of general information between the train set and the test set.

## Abbreviations

AUC: area under the curve
Ci: Composite Index
DCA: decision curve analysis
LASSO: least absolute shrinkage and selection operator
LR: logistic regression
OR: odds ratio
PICC: peripherally inserted central catheter
RF: random forest
ROC: receiver operating characteristic
SIS: Safe Infusion System
SVM: support vector machine
TOPSIS: Technique for Order Preference by Similarity to Ideal Solution
UE: unplanned extubation

## Data Availability

The data sets generated or analyzed during this study are not publicly available due to the terms of consent and permission to which the participants agreed but are available from the corresponding author upon reasonable request.
