# Supplementary material for: Risk Factors and Predictive Models for Peripherally Inserted Central Catheter Unplanned Extubation in Patients With Cancer: Prospective, Machine Learning Study
Source: J Med Internet Res. 2023 Nov 16;25:e49016. doi: 10.2196/49016 (PMC10690529; doi:10.2196/49016)
Supplement: Multimedia Appendix 1 [file jmir_v25i1e49016_app1.docx]

| **Predictor** | **Definition** |
| --- | --- |
| BMI(Body Mass Index) | BMI=weight(kg)/height(m)^2^ .According to the standards of the Chinese population, a BMI <18.5 is considered underweight, 18.5-23.9 is considered normal, 24.0-27.9 is considered overweight, and a BMI ≥28.0 is considered obese. The height and weight collected in this study were those during intubation. |
| Alcohol history | A history of persistent alcohol consumption, usually for more than 5 years, up to 40 grams per day for men and 20 grams per day for women, or a history of a large alcohol consumption in 2 weeks. |
| Mental status | It is generally defined as a disorder of consciousness dominated by altered arousal and mainly classified as drowsiness, lethargy, and unconsciousness. In this study, the presence of a non-conscious state before or 1 month after intubation was uniformly categorized as a disorder of consciousness. |
| Cooperation | Cooperation means that the study subjects have better adherence to medical treatment and can cooperate with medical practitioners to complete catheter punctures, regular catheter maintenance, receive treatment, etc. |
| Physical mobility | In this study, physical mobility was classified as normal and abnormal. Normal means that the activity is entirely independent and can move freely; abnormal means that the activity requires equipment or devices (e.g., crutches, wheelchair) or the help of others. This study refers to limb restraint or bed-ridden status caused by reduced muscle strength, increased muscle tone, hemiplegia, paralysis, burns, post-operative period of major surgery, disability, etc., in the first month after the intubation. |
| History of deep vein thrombosis | It means the study subjects had deep vein thrombosis before the current cannulation. Deep vein thrombosis refers to the abnormal clotting of blood in the deep veins, a disorder of venous reflux in the lower limbs, mainly in the braked state. |
| History of central venous placement | Refers to a previous history of central venous catheter placement before the current placement. |
| Surgical history | Refers to study subjects who had surgery within 1 month before tube insertion, excluding pathological examination-type surgeries. |
| D-D dimer concentration | The normal range is generally ≤0.5 mg/L; the data collected into the variable screening and model construction in this study were the highest values of the D-dimer test data. |
| Fibrinogen concentration | The normal range is generally 2-4 g/L, and the data collected in this study for variable screening and model construction were the highest values of fibrinogen test data. |
| Hyperosmolar drugs | Refers to strong acid and alkali drugs, hypertonic and hypotonic drugs, vasoactive drugs, antineoplastic drugs, etc. |
