# Supplementary material for: Risk Factors and Predictive Models for Peripherally Inserted Central Catheter Unplanned Extubation in Patients With Cancer: Prospective, Machine Learning Study
Source: J Med Internet Res. 2023 Nov 16;25:e49016. doi: 10.2196/49016 (PMC10690529; doi:10.2196/49016)
Supplement: Multimedia Appendix 2 [file jmir_v25i1e49016_app2.docx]

| Variables | | Train set | Test set | χ2 | P-value |
| --- | --- | --- | --- | --- | --- |
|  |  | n=2374 | n=1017 |  |  |
| Gender | Male | 1152(48.5) | 492(48.4) | 0.006 | .938 |
|  | Female | 1222(51.5) | 525(51.6) |  |  |
| Tumor type | Lung cancer | 610(25.7) | 273(26.8) | 3.79 | .88 |
|  | Thymic Cancer Breast Cancer | 576(24.3) | 252(24.8) |  |  |
|  | Gastro-colorectal cancer | 346(14.6) | 145(14.3) |  |  |
|  | Hematologic tumors | 298(12.6) | 139(13.7) |  |  |
|  | Cervical cancer | 146(6.1) | 57(5.6) |  |  |
|  | Head-neck tumors | 112(4.7) | 42(4.1) |  |  |
|  | Hepatobiliary-pancreatic tumors | 73(3.1) | 29(2.9) |  |  |
|  | Intracranial tumors | 72(3.0) | 31(3.0) |  |  |
|  | Others | 141(5.9) | 49(4.8) |  |  |
| Age | 0~11 | 39(1.6) | 21(2.1) | 5.145 | .398 |
|  | 12~18 | 48(2.0) | 30(2.9) |  |  |
|  | 19~35 | 150(6.3) | 73(7.2) |  |  |
|  | 36~59 | 1302(54.8) | 533(52.4) |  |  |
|  | 60~75 | 740(31.2) | 322(31.7) |  |  |
|  | ≥76 | 95(4.0) | 38(3.7) |  |  |
| Educational level | Illiterate primary and junior high schools | 1079(45.5) | 472(46.4) | 2.355 | .502 |
|  | Secondary and high school | 651(27.4) | 279(27.4) |  |  |
|  | College Bachelor's degree | 605(25.5) | 243(23.9) |  |  |
|  | Master's Degree Doctorate | 39(16.4) | 23(2.2) |  |  |
| BMI | ＜18.5 | 149(6.3) | 70(6.9) | 0.997 | .607 |
|  | 18.5-24.0 | 1447(61.0) | 629(61.9) |  |  |
|  | ＞24.0 | 778(32.8) | 318(31.3) |  |  |
| Alcohol history | None | 2088(88.0) | 893(87.8) | 0.014 | .906 |
|  | Yes | 286(12.0) | 124(12.2) |  |  |
| Mental status | Sobriety | 2229(93.9) | 959(94.3) | 0.207 | .649 |
|  | Blurred consciousness | 145(6.1) | 58(5.7) |  |  |
| Cooperation | Cooperative | 2214(93.3) | 948(93.2) | 0.002 | .964 |
|  | Incooperative | 160(6.7) | 69(6.8) |  |  |
| Physical mobility | Normal | 2176(91.7) | 940(92.4) | 0.565 | .452 |
|  | Abnomal | 198(8.3) | 77(7.6) |  |  |
| History of deep vein thrombosis | None | 2213(93.2) | 946(93.0) | 0.044 | .834 |
|  | Yes | 161(6.8) | 71(7.0) |  |  |
| History of central venous placement | None | 2081(87.7) | 872(85.7) | 5.297 | .151 |
|  | 1 | 170(7.2) | 94(9.2) |  |  |
|  | 2 | 89(3.7) | 33(3.2) |  |  |
|  | ≥3 | 34(1.4) | 18(1.8) |  |  |
| Diabetes | None | 2231(94.0) | 957(94.1) | 0.019 | .89 |
|  | Yes | 143(6.0) | 60(5.9) |  |  |
| Hypertension | None | 2197(92.5) | 939(92.3) | 0.047 | .828 |
|  | Yes | 177(7.5) | 78(7.7) |  |  |
| Cardiovascular disease | None | 2209(93.0) | 945(92.9) | 0.018 | .893 |
|  | Yes | 165(7.0) | 72(7.1) |  |  |
| Hyperlipidaemia | None | 2168(91.3) | 939(92.3) | 0.942 | .332 |
|  | Yes | 206(8.7) | 78(7.7) |  |  |
| Surgical history | None | 1436(60.5) | 636(62.5) | 1.257 | .262 |
|  | Yes | 938(39.5) | 381(37.4) |  |  |
| D-D dimer concentration | ≤0.5 | 1966(82.8) | 826(81.2) | 1.245 | .265 |
|  | ＞0.5 | 408(17.2) | 191(18.8) |  |  |
| Fibrinogen concentration | Lower | 71(3.0) | 30(2.9) | 0.372 | .83 |
|  | Normal | 1746(73.5) | 758(74.5) |  |  |
|  | Higher | 557(23.5) | 229(22.5) |  |  |
| Radiotherapy treatment | None | 2145(90.4) | 932(91.6) | 1.406 | .236 |
|  | Yes | 229(9.6) | 85(8.4) |  |  |
| Targeted therapy | None | 1105(46.5) | 458(45.0) | 0.655 | .418 |
|  | Yes | 1269(53.5) | 559(55.0) |  |  |
| Surgical treatment | None | 1858(78.3) | 815(80.1) | 1.497 | .221 |
|  | Yes | 516(21.7) | 202(19.9) |  |  |
| Anticoagulation | None | 2203(92.8) | 958(94.2) | 2.212 | .137 |
|  | Yes | 171(7.2) | 59(5.8) |  |  |
| Chemotherapy treatment | None | 318(13.4) | 146(14.4) | 0.557 | .455 |
|  | Yes | 2056(86.6) | 871(85.6) |  |  |
| Hyperosmolar drugs | None | 1083(45.6) | 483(47.5) | 1.005 | .316 |
|  | Yes | 1291(54.4) | 534(52.5) |  |  |
| Limb on side of placement | Left upper extremity | 1158(48.8) | 543(53.4) | 7.708 | .052 |
|  | Right upper extremity | 1162(48.9) | 446(43.9) |  |  |
|  | Left lower extremity | 23(1.0) | 13(1.3) |  |  |
|  | Right lower extremity | 31(1.3) | 15(1.5) |  |  |
| Puncture method | Blind | 75(3.2) | 33(3.2) | 5.287 | .071 |
|  | Blind-MST | 111(4.7) | 67(6.6) |  |  |
|  | B ultrasound-MST | 2188(92.2) | 917(90.2) |  |  |
| Puncture times | 1 | 2223(93.6) | 958(94.2) | 0.383 | .536 |
|  | Many times | 151(6.4) | 59(5.8) |  |  |
| Catheter gauge | 1.9Fr | 4(0.2) | 4(0.4) | 7.562 | .056 |
|  | 3Fr | 66(2.8) | 21(2.1) |  |  |
|  | 4Fr | 2185(92.0) | 922(90.7) |  |  |
|  | 5Fr | 119(5.0) | 70(6.9) |  |  |
| Catheter lumen | Single-chamber | 2103(88.6) | 918(90.3) | 2.069 | .15 |
|  | Double-chamber | 271(11.4) | 99(9.7) |  |  |
| Catheter material | Silicone | 1004(42.3) | 455(44.7) | 1.74 | .187 |
|  | Polyurethane | 1370(57.7) | 562(55.3) |  |  |
| Presence of valve | None | 1020(43.0) | 440(43.2) | 0.026 | .872 |
|  | Yes | 1354(57.0) | 577(56.7) |  |  |
| Whether high-pressure resistant catheter | None | 1864(78.5) | 800(78.7) | 0.791 | .374 |
|  | Yes | 510(21.5) | 217(21.3) |  |  |
